# Room-temperature continuous-wave electrically pumped InGaN/GaN quantum well blue laser diode directly grown on Si

**DOI:** 10.1038/s41377-018-0008-y

**Published:** 2018-06-13

**Authors:** Yi Sun, Kun Zhou, Meixin Feng, Zengcheng Li, Yu Zhou, Qian Sun, Jianping Liu, Liqun Zhang, Deyao Li, Xiaojuan Sun, Dabing Li, Shuming Zhang, Masao Ikeda, Hui Yang

**Affiliations:** 1 Key Laboratory of Nanodevices and Applications, Suzhou Institute of Nano-Tech and Nano-Bionics (SINANO), Chinese Academy of Sciences (CAS), Suzhou, 215123 China; 2 School of Nano Technology and Nano Bionics, University of Science and Technology of China, Hefei, 230026 China; 3 Accelink Technologies Co., Ltd, Wuhan, 430205 China; 4 State Key Laboratory of Luminescence and Applications, Changchun Institute of Optics Fine Mechanics and Physics (CIOMP), CAS, Changchun, 130033 China

## Abstract

Current laser-based display and lighting applications are invariably using blue laser diodes (LDs) grown on free-standing GaN substrates, which are costly and smaller in size compared with other substrate materials.^1–3^ Utilizing less expensive and large-diameter Si substrates for hetero-epitaxial growth of indium gallium nitride/gallium nitride (InGaN/GaN) multiple quantum well (MQW) structure can substantially reduce the cost of blue LDs and boost their applications. To obtain a high crystalline quality crack-free GaN thin film on Si for the subsequent growth of a blue laser structure, a hand-shaking structure was formed by inserting Al-composition step down-graded AlN/Al_x_Ga_1−x_N buffer layers between GaN and Si substrate. Thermal degradation in InGaN/GaN blue MQWs was successfully suppressed with indium-rich clusters eliminated by introducing hydrogen during the growth of GaN quantum barriers (QBs) and lowering the growth temperature for the p-type AlGaN/GaN superlattice optical cladding layer. A continuous-wave (CW) electrically pumped InGaN/GaN quantum well (QW) blue (450 nm) LD grown on Si was successfully demonstrated at room temperature (RT) with a threshold current density of 7.8 kA/cm^2^.

An InGaN/GaN QW blue LD as a key device component holds great potential for a wide range of applications^4^, including laser displays, laser-based automotive lighting, visible light communication^5^, undersea wireless communication^6^, and high-density data storage^7^. Today, most InGaN/GaN QW blue LDs are homo-epitaxially grown on expensive 2-inch free-standing GaN substrates^3^, which leads to LDs costing orders of magnitude higher than light-emitting diodes (LEDs) grown on hetero-epitaxial substrates (such as sapphire, SiC, or silicon). In addition, free-standing GaN substrates often suffer from non-uniformity in the offcut angle, defect density, and/or residual stress, affecting process reproducibility and production yield^8^. By contrast, Si substrates show several advantages in terms of wafer size, material cost, and uniformity, as well as the well-developed automotive processing line. By replacing costly and small-size GaN free-standing substrates with cost-effective and large-diameter Si substrates, the cost of InGaN/GaN QW blue LDs can be reduced to the same level as LEDs, which will lead to further applications of LDs. Here, we demonstrate a continuous-wave (CW) electrically pumped InGaN/GaN QW blue LD grown on a Si substrate operating at room temperature (RT).

GaN growth on Si typically involves a large mismatch in lattice constant and a huge misfit in coefficient of thermal expansion (CTE), which typically lead to a very high threading dislocation density (TDD) (10^9^–10^10^ cm^−2^) and a significant tensile stress or, as is often the case, the formation of a micro-crack network, respectively^9^. Furthermore, edge-emitting LDs require low-refractive-index optical cladding layers for optical field confinement; however, the growth of AlGaN-containing optical cladding layers on GaN induces additional tensile stress. Therefore, defect control and stress engineering are critical for the realization of electrically pumped InGaN/GaN LDs grown on a Si substrate^10^.

These issues can be addressed by inserting a stack of Al-composition step down-graded AlN/Al_x_Ga_1−x_N multi-layers between Si and GaN^10–12^, as shown in Fig. 1. The compressive strain built up by the positive lattice mismatch in the GaN epitaxial film on the Si substrate was utilized to compensate for the tensile stress caused by the CTE mismatch during the cool-down period. Moreover, it was also found that threading dislocations (TDs) are more likely to incline and annihilate each other, especially at interfaces facilitated by the aforementioned compressive strain, resulting in a high crystalline quality GaN film that is epitaxially grown on a Si substrate^12–14^. The crystalline quality of the GaN film grown on Si was evaluated by double crystal X-ray rocking curve measurements (Fig. 2). The linewidths for both of the GaN (0002) and (1012) X-ray diffraction peaks were approximately 260 arcsec, corresponding to a low TDD (~ 5 × 10^8^ cm^−2^). The limited difference between these two linewidths indicated a low density of edge type TDs^15,16^, which often serve as non-radiative recombination centers and, hence are more deterious to the internal quantum efficiency compared to screw and mixed type TDs^17^.

**Fig. 1:**
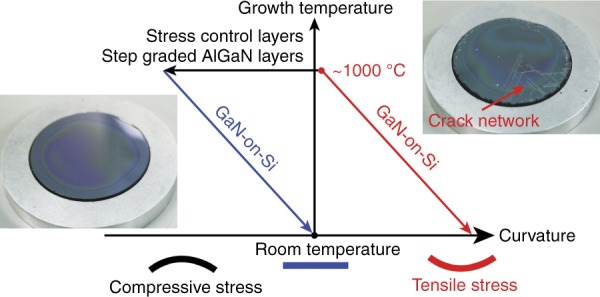
Schematic diagram of the stress control for the hetero-epitaxy growth of crack-free GaN thin film on a Si substrate with Al-composition step down-graded AlN/AlGaN buffer layers

**Fig. 2:**
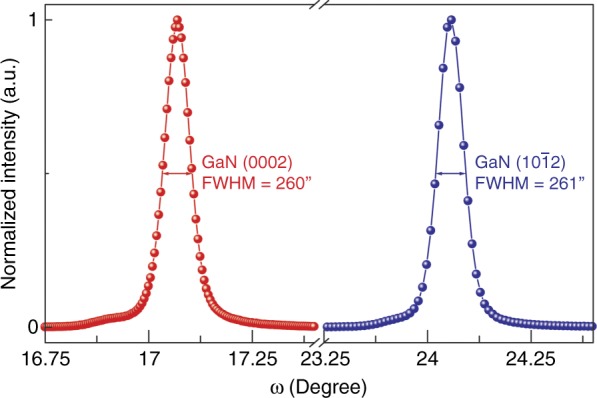
Double crystal X-ray rocking curves for the GaN (0002) and (1012) diffraction peaks for a GaN film grown on a Si substrate. The value of full width at half maximum (FWHM) is labeled

We have previously successfully demonstrated InGaN/GaN QW violet LDs directly grown on Si substrate, lasing at a wavelength of 413 nm^10^. To further expand applications for laser displays and laser lighting, blue LDs with emission lasing wavelength longer than 450 nm are highly desirable. However, growing an InGaN/GaN QW blue LD structure on a Si substrate is more challenging: the longer the emission wavelength, the smaller the difference in refractive index between III-nitride materials^18^. Therefore, both the In composition in the InGaN waveguide layers and the Al composition in the AlGaN optical cladding layers must be increased to obtain a sufficient optical confinement for the realization of blue lasing^19^. However, thick InGaN waveguide layers, which are beneficial for optical confinement, often lead to strain relaxation. Hence, InGaN and GaN compound waveguide layers were used for both optical confinement and strain control (Supplementary Fig. S1). Moreover, the inserted GaN waveguide layers also served as a transition layer between the InGaN waveguide and the AlGaN optical cladding layer to mitigate the large lattice mismatch and improve the material quality (Fig. 3b). Simulation of the optical field distribution in the GaN-on-Si blue laser structure is shown in Supplementary Fig. S1. It is noted that most of the stimulated emission photons were confined in the active region, avoiding strong absorption by the Si substrate, which was evidenced by the near-field patterns (Supplementary Fig. S2). According to scanning transmission electron microscopy (STEM) cross-sectional images (Fig. 3), sharp interfaces for the individual layers indicated a high quality for the as-grown blue LD structure on Si.

**Fig. 3:**
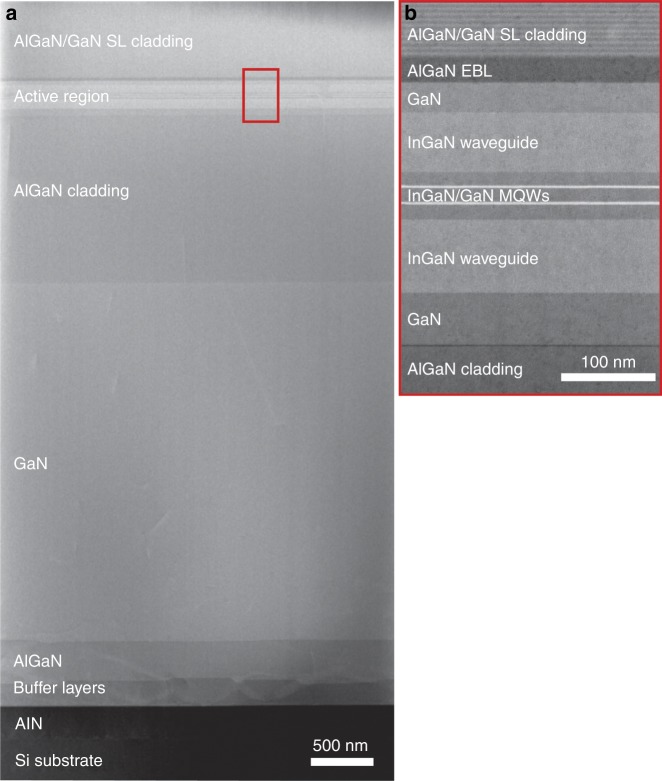
Cross-sectional STEM images for InGaN/GaN QW blue LD structure grown on a Si substrate. **a** Cross-sectional STEM image for the whole InGaN/GaN QW blue LD structure grown on a Si substrate. **b** Enlarged image of the InGaN/GaN MQW active region marked with red rectangle in a

On the other hand, a longer lasing wavelength also means a higher In composition in the MQWs, and threshold current density often increases dramatically with the In composition in MQWs^20^, especially when the dislocation density is relatively high (~5 × 10^8^ cm^−2^). Furthermore, the InGaN/GaN MQWs in blue LDs often suffer from thermal degradation due to the extended thermal process during the deposition of the upper AlGaN optical cladding layer at high temperature. Thermal degradation in InGaN/GaN MQWs in blue LDs was often observed through the formation of dark spots under micro-photoluminescence (micro-PL) imaging (Fig. 4a). The dark spots were related to thermally degraded regions in the MQWs, where metallic indium precipitates and voids are found^21^. It is generally believed that the precipitation of metallic indium and void formation originate from the decomposition of a fraction of indium-rich clusters in the QW/quantum barrier (QB) interfaces or the QWs during the growth of the p-type optical cladding layer at high temperature^22,23^. Indium-rich clusters can form due to indium segregation^24^, indium accumulation around the TDs^25^, or indium-rich trench defects, as reported recently^26,27^. It is crucial to eliminate such defects and ensure that the InGaN/GaN blue MQWs are sufficiently robust to survive the extended thermal process during the upper AlGaN cladding layer growth.

**Fig. 4:**
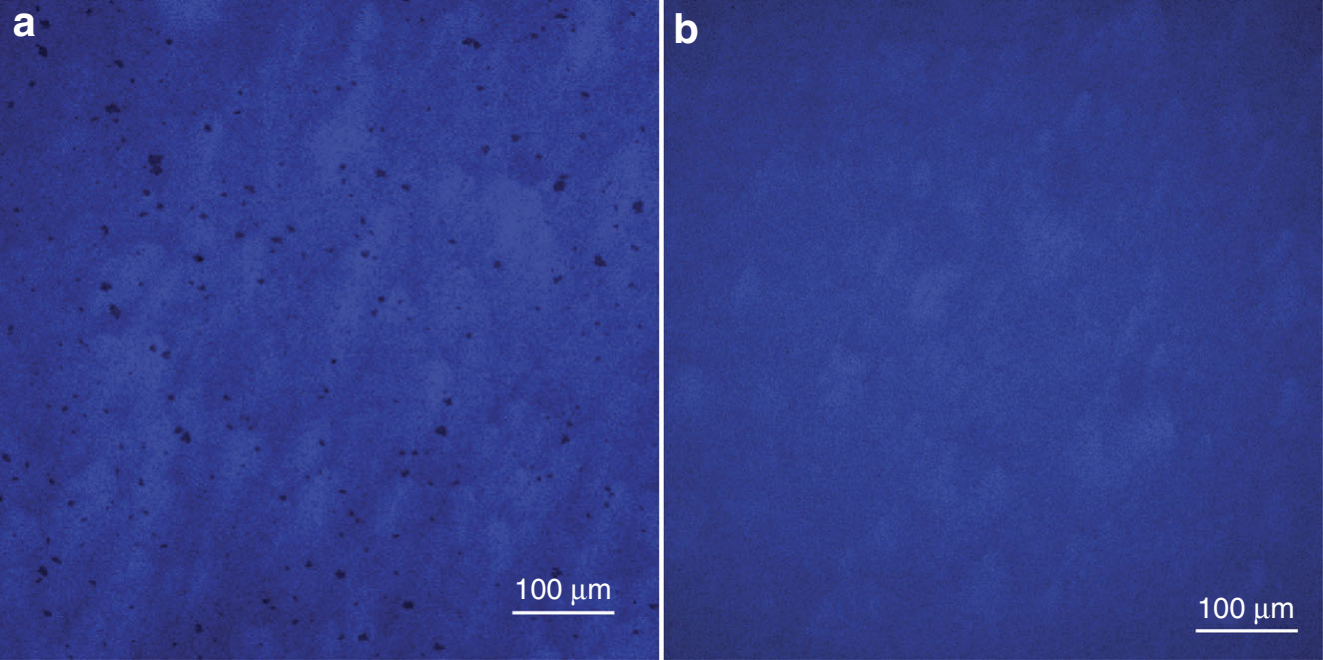
Thermal degradation in InGaN/GaN MQWs in blue LDs on Si. Micro-PL images of GaN-on-Si blue LD active region **a** with and **b** without thermal degradation

To avoid thermal degradation in InGaN/GaN blue MQWs, we used H_2_ to eliminate In-rich clusters and also reduced the growth temperature for the upper AlGaN cladding layer^28^. Thermal degradation can be mitigated by reducing the thermal budget, which was realized by lowering the growth temperature of the thick p-AlGaN cladding layer from 978 to 970 °C. It should be noted that a further reduction in growth temperature for the p-AlGaN material can cause a significant increase in series resistance and device operating voltage because of the unintentional incorporation of carbon impurities in the p-type materials^29^. More importantly, In-rich clusters must be removed to make the InGaN/GaN blue MQWs more robust. Since H_2_ can effectively etch away indium material from the growth surface, we intentionally introduced 600 sccm of H_2_ into the carrier gas (corresponding to only 3% of the total gas flow rate) during the GaN QBs growth after the QW cap layer was deposited. The H_2_ source was switched off prior to the growth of the InGaN QW, ensuring no negative effect on indium incorporation during the InGaN QW growth. Meanwhile, a dual-temperature growth scheme was adopted when growing InGaN/GaN blue MQWs. The growth temperature of GaN QBs was 80 °C higher than that of InGaN QWs, which can also assist in the elimination of indium segregation, indium accumulation, and indium-rich trench defects. As a result, thermal degradation of the InGaN/GaN blue MQWs in the LD structure was effectively suppressed, as evidenced by the complete elimination of dark spots in the micro-PL imaging of the blue LD structure grown on Si (Fig. 4b).

The as-grown InGaN/GaN blue LD epitaxial wafer was fabricated into edge-emitting LDs (Fig. 5a, b) with a ridge width of 4 μm and a cavity length of 400 μm (see Materials and methods). The characteristics of the as-fabricated InGaN/GaN blue LD grown on Si under a CW electrical injection at RT are shown in Fig. 5c–f. The lasing wavelength was 450.3 nm with a threshold current of 125 mA, corresponding to a threshold current density of 7.8 kA/cm^2^. Fig. 5c shows the electroluminescence (EL) spectra of the LD under a CW electrical pumping below (100 mA) and above (130 mA) the threshold. When the injection current rose up from 100 to 130 mA, the peak wavelength of the EL spectra was blue-shifted from 451.5 to 450.3 nm, because of the screening of the quantum-confined Stark effect. At the same time, the full width at half maximum (FWHM) of the EL spectra quickly decreased to 0.46 nm. The far-field patterns (FFPs) of the edge emission with a CW injection above and below the threshold current are shown in Fig. 5d, e, respectively. As a result of the asymmetric optical confinement in the longitudinal and latitudinal directions, an elliptical FFP (Fig. 5d) was observed elongated along the growth direction. The light–current–voltage (L-I-V) characteristics for the blue LD grown on Si under a CW electrical injection at RT are shown in Fig. 5f. The light output power increased much more rapidly when the injection current went beyond the threshold current of 125 mA. The relatively high operating voltage for the as-fabricated LD was mainly due to the relatively large resistance of the p-type GaN/AlGaN superlattice optical cladding layer grown at a relatively low temperature with an unintentional doping of carbon impurities^29^, which, as compensation centers, greatly reduced the hole concentration. About 68% of the fabricated LDs could lase and the statistical results about the threshold current of the as-produced blue LDs grown on Si are shown in Supplementary Fig. S3, which presents a decent yield for the device processing.

**Fig. 5:**
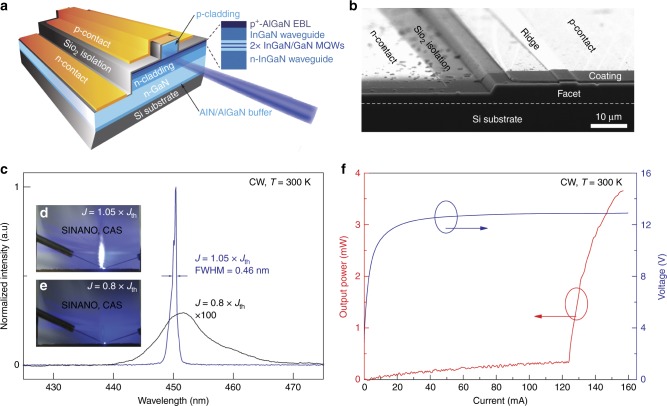
Schematic architecture, scanning electron microscope (SEM) image and characteristics of blue GaN-on-Si LD. **a** Schematic structure of a blue LD directly grown onto a Si substrate. **b** Bird-view SEM image of a blue LD grown on Si. **c** EL spectra and FFPs of a blue LD grown on Si under a CW electrical pumping at RT. FFPs were observed **d** above and **e** below the threshold current by placing a sheet of paper in front of the LD front facet. **f** L-I-V characteristics of the InGaN/GaN blue LD measured under a CW current injection at RT

It should be noted that the light output power under CW current injection at RT was measured following simple packaging with indium soldering. The slope efficiency of these devices shows a clear decrease after increasing the current injection to 130 mA (Fig. 5f). This reduced slope efficiency of the blue LD grown on Si under CW electrical injection was caused by a self-heating problem because of the relatively high threshold current density and the relatively high operation voltage of the as-fabricated devices (Fig. 5f). The severe self-heating problem not only affected the LD performance but also shortened its lifetime (less than 1 min), especially under CW operation. The self-heating problem can be mitigated by improving heat dissipation with a better package and by decreasing the threshold current and the operation voltage via optimization of the epitaxial structure and the growth conditions, as well as the LD fabrication process. In particular, the operating voltage can be greatly reduced by lowering the resistance of the p-type GaN/AlGaN superlattice optical cladding layer, which can be grown at a relatively lower temperature, but at a slower growth rate to suppress unintentional doping of carbon^29^. It is believed that the lifetime of the as-fabricated blue LDs grown on Si was also limited by the high TDD^30^. According to the previous reports, the lifetime of InGaN-based LDs can be improved from a few seconds^31^ to 300 h^32^, when the threshold current density was reduced from 9 to 4.2 kA/cm^2^, and the operation voltage was lowered from 8 to 4 V. In addition, it has been reported that the lifetime of InGaN-based LDs can be further extended to more than 10,000 h when the TDD in the LD structure is reduced from 10^8^ down to 10^6^ cm^−2^ via an epitaxial lateral overgrowth of GaN^30^. For blue LDs grown on Si, lowering of the operation voltage to approximately 4 V and reduction of the density of TDs from 10^8^ to 10^6^ cm^−2^ in GaN grown on Si through epitaxial lateral overgrowth is expected to decrease the threshold current density to less than 4 kA/cm^2^, with the lifetime possibly reaching a few hundred or even thousands of hours.

In conclusion, we have demonstrated a CW operation of InGaN/GaN QW blue LDs grown on a Si substrate at RT. The threshold current density was 7.8 kA/cm^2^ and the peak wavelength for the blue LD grown on Si was 450.3 nm. These results highlight a promising technical approach for manufacturing InGaN/GaN blue LDs at substantially lower cost by using large-size Si as the epitaxial substrate. With further improvement in the lifetime of devices by reducing the threshold current and the operating voltage, together with reduction in the TD density by using epitaxial lateral overgrowth, GaN-on-Si technology holds great potential for the commercialization of InGaN-based blue LDs fabricated using cost-effective, large-diameter Si substrates, further boosting wide applicability.

A commercial available metal–organic chemical vapor phase deposition system was employed for the growth of InGaN/GaN QW blue LD structure on a Si(111) substrate. Nitrogen and hydrogen served as carrier gases. Monosilane and bisethylcyclopentadienylmagnesium were adopted as the n- and p-type dopants, respectively. Trimethylaluminum, trimethylindium, trimethylgallium, and ammonia were utilized as precursors for aluminum, indium, gallium, and nitrogen, respectively. A 2-inch Si(111) substrate was firstly thermally cleaned before the growth of the hand-shaking buffer, which consisted of a 280-nm-thick AlN nucleation layer, a 180-nm-thick Al_0.35_Ga_0.65_N layer, and a 310-nm-thick Al_0.17_Ga_0.83_N layer. A 3-μm-thick n-GaN layer was then deposited on top of the hand-shaking buffer. Afterwards, an InGaN/GaN QW blue LD structure was then grown, which consisted of a 1.4-μm-thick n-type Al_0.06_Ga_0.94_N optical cladding layer, a 55-nm-thick n-type GaN transition layer, a 75-nm-thick n-type In_0.04_Ga_0.96_N waveguide layer, two pairs of 2.5-nm-thick undoped In_0.15_Ga_0.85_N QW and 15-nm-thick undoped GaN QB layers, a 60-nm-thick undoped In_0.035_Ga_0.965_N waveguide layer, a 30-nm-thick undoped GaN transition layer, a 20-nm-thick p-type Al_0.2_Ga_0.8_N electron blocking layer, one hundred pairs of 3-nm-thick p-type Al_0.14_Ga_0.86_N and 3-nm-thick p-type GaN superlattice optical cladding layers, and a 30-nm-thick p-type GaN contact layer.

The as-grown blue LD epitaxial wafer was fabricated into edge-emitting LDs with a ridge width of 4  μm^10^. The cavity length was 400  μm, which was formed by facet cleavage. Three and seven pairs of quarter-wave TiO_2_/SiO_2_ reflective coatings were deposited on the front and the back facets, respectively. The LD device structure was lateral, with both the p- and the n-contact pads on the same side of the device, as shown in Fig. 5a, b.

An optical power meter (Thorlabs PM121D) was used to measure the output power under CW electrical pumping. A fiber optic spectrometer (IdeaOptics FX4000) was utilized to measure the electrical luminescence spectra of the LDs. A PANalytical X’Pert Pro MRD high-resolution X-ray diffractometer was employed for the double-crystal X-ray rocking curve measurements. A Hitachi HD-2700 field emission (FE)-STEM operated at 200  kV and an FEI Quanta 400 FEG thermal field emission SEM operated at 10 kV were used for taking the STEM and the SEM images, respectively.

## Electronic supplementary material

SUPPLEMENTAL MATERIAL(DOCX 619 kb)
