# Supplementary material for: Room-temperature continuous-wave electrically pumped InGaN/GaN quantum well blue laser diode directly grown on Si
Source: Light Sci Appl. 2018 Jun 13;7:13. doi: 10.1038/s41377-018-0008-y (PMC6106987; doi:10.1038/s41377-018-0008-y)
Supplement: Supplementary file 1 — SUPPLEMENTAL MATERIAL(DOCX 619 kb) [file 41377_2018_8_MOESM1_ESM.docx]

**Title: Room-temperature continuous-wave electrically pumped InGaN/GaN quantum well blue laser diode directly grown on Si**

**Authors:** Yi Sun^1,2,3^, Kun Zhou^1^, Meixin Feng^1,2^, Zengcheng Li^1^, Yu Zhou^1,2^, Qian Sun^1,2^*, Jianping Liu^1^*, Liqun Zhang^1^, Deyao Li^1^, Xiaojuan Sun^4^, Dabing Li^4^, Shuming Zhang^1^, Masao Ikeda^1^, and Hui Yang^1^

**Affiliations:**

^1^Key Laboratory of Nanodevices and Applications, Suzhou Institute of Nano-Tech and Nano-Bionics (SINANO), Chinese Academy of Sciences (CAS), Suzhou 215123, P. R. China

^2^Suzhou Institute of Nano-Tech and Nano-Bionics, Chinese Academy of Sciences, Nanchang, 330200, P. R. China

^3^Accelink Technologies Co., Ltd., Wuhan 430205, P. R. China

^4^State Key Laboratory of Luminescence and Applications, Changchun Institute of Optics Fine Mechanics and Physics (CIOMP), CAS, Changchun 130033, P. R. China

*Correspondence to: [qsun2011@sinano.ac.cn](mailto:xxxxx@xxxx.xxx) (Q. S); jpliu2010@sinano.ac.cn (J. L)

This file contains:

1. Optical field distribution in the GaN-on-Si blue laser structure

2. Near-field pattern (NFP) distribution of the GaN-on-Si blue laser diode

3. The statistical measurement results of the threshold current of the as-fabricated devices

**1. Optical field distribution in the GaN-on-Si blue laser structure**

To obtain enough optical confinement, InGaN and GaN compound waveguide layers were used. Figure S1 showed the optical field distribution in the GaN-on-Si blue laser structure. The optical confinement in the InGaN blue QWs was 1.36%.


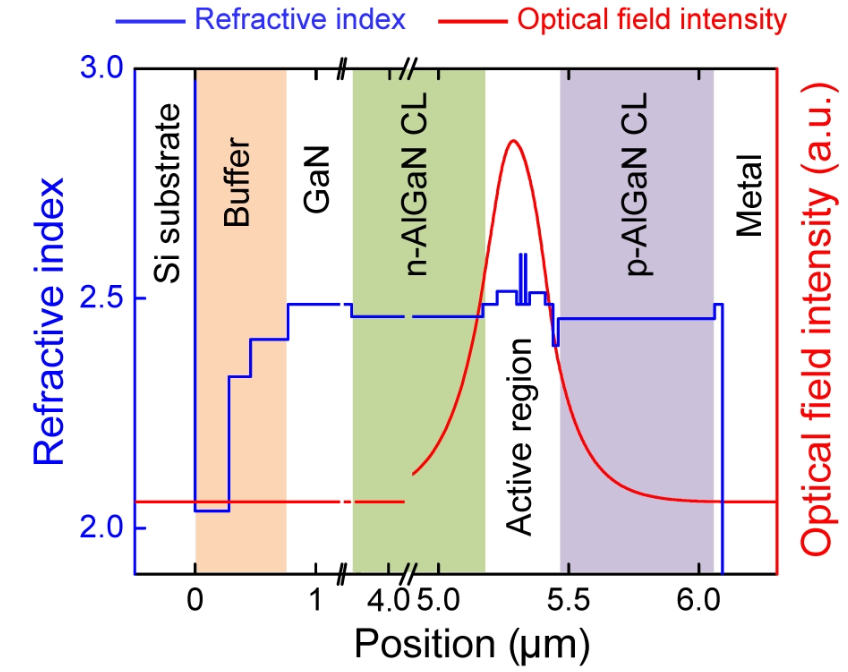


Figure S1. The optical field distribution in the GaN-on-Si blue laser structure.

**2. NFP distribution of the GaN-on-Si blue laser diode**

Cross-sectional NFPs of one as-fabricated GaN-on-Si blue LD after facet coating were observed by using an optical microscope from the front facet at a current injection below (Fig. S2, a and b) and above the threshold current (Fig. S2c). When the injection current reached the threshold current, strong stimulated emissions were clearly observed from the front facet under the ridge structure. It should be noted that for the as-fabricated GaN-on-Si LDs, the NFPs did not extend to the Si substrate under any current injection, indicating that with the presence of the optical cladding and waveguide layers, the strong absorbing Si substrate did not affect the stimulated emission.

**
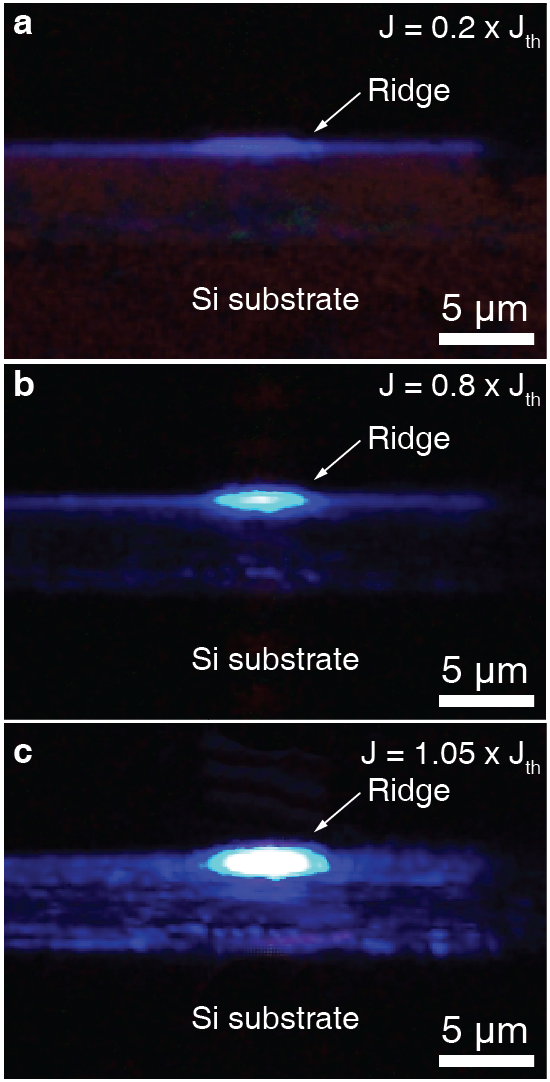
**

Figure S2.NFPs of a GaN-on-Si blue LD under an injection current of **a.** 0.2J_th_, **b.** 0.8J_th_, and **c.** 1.05J_th_.

**3. The statistical measurement results of the threshold current of the as-fabricated devices**

In order to evaluate the yield and the reproducibility, we measured 400 devices on forty bars of as-fabricated GaN-on-Si LDs from the same epitaxial wafer. Figure S3 shows the statistical results for the threshold current of the GaN-on-Si LDs. The measurement was performed under pulsed current injection (pulse width of 1 μs and repetition rate of 10 kHz). 272 devices (68%) of the measured GaN-on-Si LDs could lase. The lowest, average and medium threshold current for the as-fabricated GaN-on-Si LDs were 95, 257 and 190 mA, corresponding to a threshold current density of 6, 16 and 11.9 kA/cm^2^, respectively. These results exhibit a decent yield of the as-fabricated GaN-on-Si blue LDs.


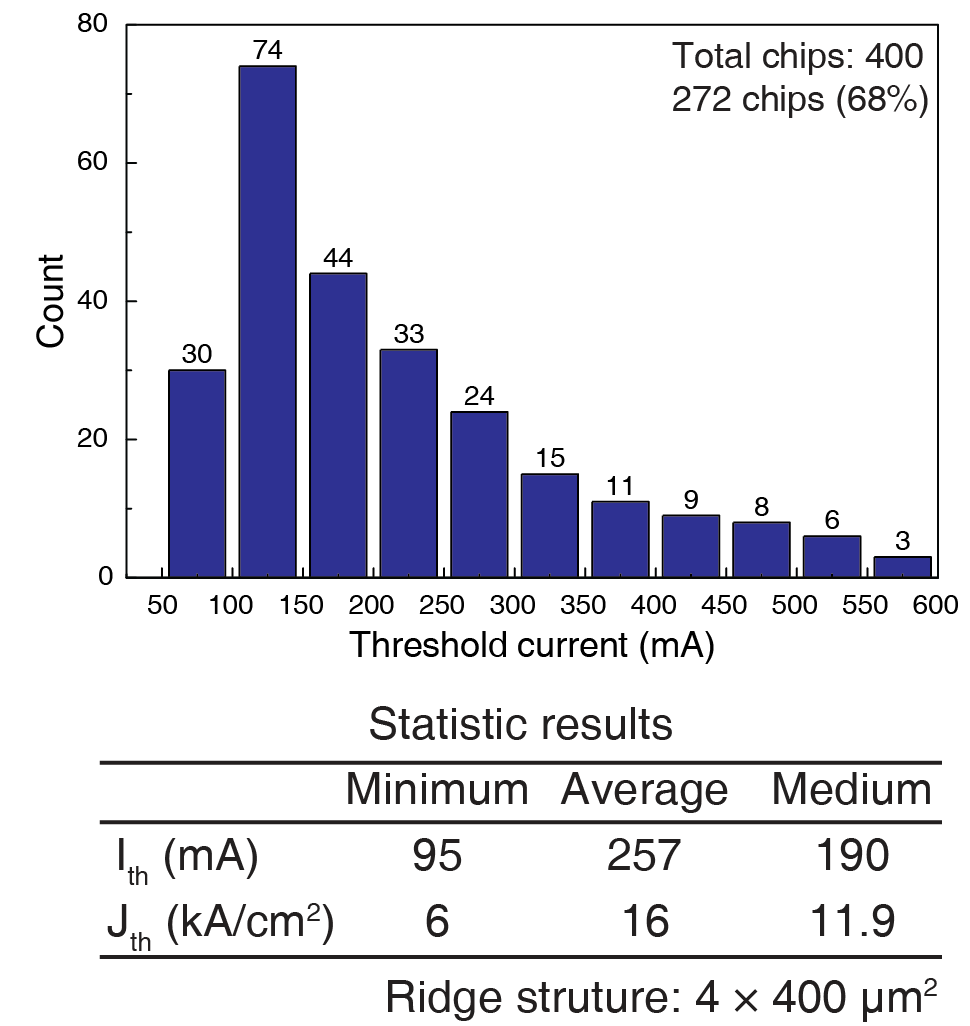


Figure S3. Statistical results of the threshold current of the as-fabricated GaN-on-Si blue LDs.
